# Influence of Calcined Flue Gas Desulfurization Gypsum and Calcium Aluminate on the Strength and AFt Evolution of Fly Ash Blended Concrete under Steam Curing

**DOI:** 10.3390/ma14237171

**Published:** 2021-11-25

**Authors:** Yueran Zhang, Heng Zhang, Xiong Zhang

**Affiliations:** 1 Key Laboratory of Advanced Civil Engineering Materials of the Ministry of Education, School of Materials Science and Engineering, Tongji University, Shanghai 201804, China; 1610978@tongji.edu.cn (Y.Z.); 1732840@tongji.edu.cn (H.Z.); 2 CCCC Shanghai Harbour Engineering Design & Research Institute Co., Ltd., Shanghai 200032, China

**Keywords:** calcined FGD gypsum, active calcium aluminate, composite cementitious system, strength, volume stability

## Abstract

In order to improve the early strength of fly ash blended cement concrete under steam curing conditions, fly ash was partly substituted by calcined flue gas desulfurization (FGD) gypsum and active calcium aluminate. The effect of the composition and curing condition on the workability, mechanical property, and volume stability was systematically evaluated. The variety of hydration products and the evolution was determined by XRD to explore the formation kinetic of ettringite. Results show that the addition of calcined FGD gypsum and active calcium aluminate is able to improve the early compressive strength but using more FGD gypsum and a high sulfur aluminum ratio leads to a reduction in compressive strength from 28 to 90 days due to the increment of ettringite and crystallization of dihydrate gypsum. Both the free expansion ratio and limited expansion exhibited a continuous increasement with time, especially in the first 14 days of testing. Cracks were not observed on the surface of samples immersed in water for a year. The improvement of strength and shrinkage resistance is mainly due to the formation of ettringite generated before 14 days and the precipitation was highly limited from 14 to 28 days. Moreover, the characteristic peak of gypsum appeared after 28 days, indicating the conversion of partial of calcined FGD gypsum. The work presented here provides a new solution for improving the early strength of fly ash concrete without reducing the later strength and consuming extra energy.

## 1. Introduction

Flue gas desulfurization (FGD) gypsum is an industrial by-product generated during the flue gas desulfurization process in coal-fired power plants. According to the American Coal Ash Association, FGD gypsum production rates have increased from ~11 million metric tons (MT) in 2006 to ~29 million MT in 2016. However, this increase in FGD gypsum generation has not been accompanied with a similar increase in its utilization, with utilization rates decreasing from 79% in 2006 to 57% in 2016 [1]. With the rapid development of industries, FGD gypsum has become one of the bulk industrial solid wastes, and triggers many local environmental problems in China [2]. It is estimated that the output has reached up to 80 million tons annually in China. This has led to FGD gypsum accumulation, creating the need for extra capacity in landfills. To reduce the amounts of disposed FGD gypsum, an increase in its utilization rate through new applications is necessary. The recent beneficial use of FGD gypsum is in wallboard production, concrete/cement and asphalt production, CaCO3 production, and the production of calcium sulfate. Untreated FGD gypsum is mainly composed of calcium sulfate and can be recycled in main industrial fields [3]. The direct heaping of FGD in the natural environment also results in land occupancy and environmental pollution [4]. At present, the industrial utilization of desulfurized gypsum has become one of the main research directions of scholars. Comprehensive utilization of desulfurized gypsum conforms to the concept of green and low-carbon development. Therefore, full research on the comprehensive performance of desulfurized gypsum in different systems will greatly promote its industrial application. To solve the problem of the low early strength of the fly ash-desulfurized gypsum system, this study innovatively uses sulfate to increase its early strength without shrinking late strength, and successfully applies desulfurized gypsum to prepare early strength and high-strength mortar systems.

Many investigations were devoted to resourceful utilization of FGD gypsum in construction materials. The results of physical chemistry and morphological characterization of both FGD gypsum and natural gypsum in Portland cement products showed a material of high purity, calcium sulfate dehydrate for natural gypsum, and higher percentages of bassanite and hannebachite with low concentrations of impurities for FGD gypsum [5]. In the presence of FGD gypsum, the setting times are much slower than those of pastes in the absence of FGD gypsum. The combination of FGD gypsum and slag powder provides synergistic benefits above that of slag powder alone. The addition of FGD gypsum provides benefit by promoting ettringite formation and forms a compact microstructure, increasing the compressive strength and reducing the drying shrinkage of cement mortar and concrete [6]. The use of hemihydrate (FGDG) would accelerate the formation of phases, based on the heat patterns [7]. The calcination-treated FGD gypsum would not influence the type and morphology hydration products of the calcium sulfur aluminate cement-fly ash-FGD gypsum system, and the calcination of FGD gypsum is able to promote the hydration degree of fly ash especially in the presence of lime, making the hardened slurry more compact [8,9,10].

The above research shows that the application of desulfurized gypsum in concrete systems will help improve the overall performance of concrete, realize the industrial utilization of desulfurized gypsum, and turn desulfurized gypsum into treasure, which is in line with the green and low-carbon development concept. However, adding FGD gypsum to the system will delay the setting time of the mortar and will have a bad influence on the early strength [11]. If the amount of FGD gypsum is too large, it will cause larger volume expansion in the later stage, which will cause concrete cracking and other phenomena. Therefore, an appropriate amount of gypsum is very important for the development of system strength and volume stability [12]. However, due to the low hydration activity of fly ash, the heat release during the entire hydration process is small, and the hydration reaction is slow, resulting in the problem of low early strength in the system of fly ash and desulfurized gypsum [13]. In our previous work, the feasibility of using the calcined FGD gypsum and fly ash as the supplementary cementitious materials in the manufacture of precast concrete was confirmed from the aspect of the hydration and mechanical properties. There is a limitation that the early compressive strength development was delayed. Because aluminate cement has early strength characteristics, the CA and C_2_A contained in it can undergo a hydration reaction when it meets with water. At the same time, it can provide more aluminate ions and react with sulfate ions in gypsum to generate more AFt. Then, the AFt overlaps each other to form a network structure, and promote the improvement of early strength. In order to promote the early strength development, a small amount of calcium aluminate was added to compensate the low activity of fly ash, and blended with a calcined FGD gypsum as a ternary system for the precast concrete manufacturing. The effect of the composition and curing condition on the workability, mechanical property, and volume stability was systematically evaluated. The variety of hydration products was determined by XRD to explore the formation kinetic of ettringite.

## 2. Experiments

### 2.1. Materials

The raw materials employed in this work were P.II.52.5 Portland cement from Nanjing China, FGD gypsum from Xiamen China, fly ash from Sichuan thermal power plant, and calcium aluminate cements with 59% activated alumina from Kerneos Co., Ltd. (Tianjin, China). The chemical compositions of P.II.52.5 Portland cement, FGD gypsum, and fly ash are shown in Table 1.

**Figure 1:**
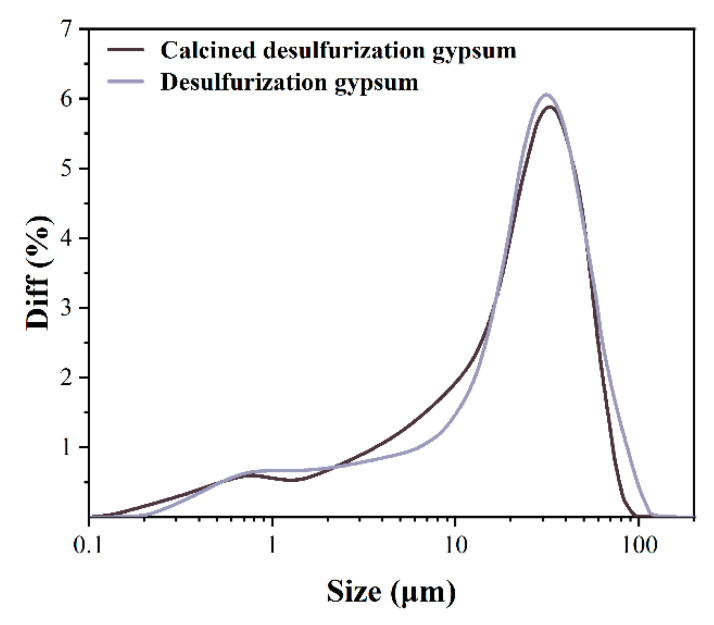
Particle size distribution of FGD gypsum.

The FGD gypsum was firstly calcined in the DRY-36 high-temperature industrial furnace at 800 °C for 1 h, and then cooled to room temperature within the furnace. The effect of calcination on the fineness and chemical composition was evaluated. As shown in Figure 1, the calcination tends to reduce the particle size. More than 47% of calcined FGD gypsum is finer than 20 μm, and around 52% of particles are 20~80 μm. The raw FGD gypsum is coarser and more than 56% is in the range between 20 and 80 μm.

**Figure 2:**
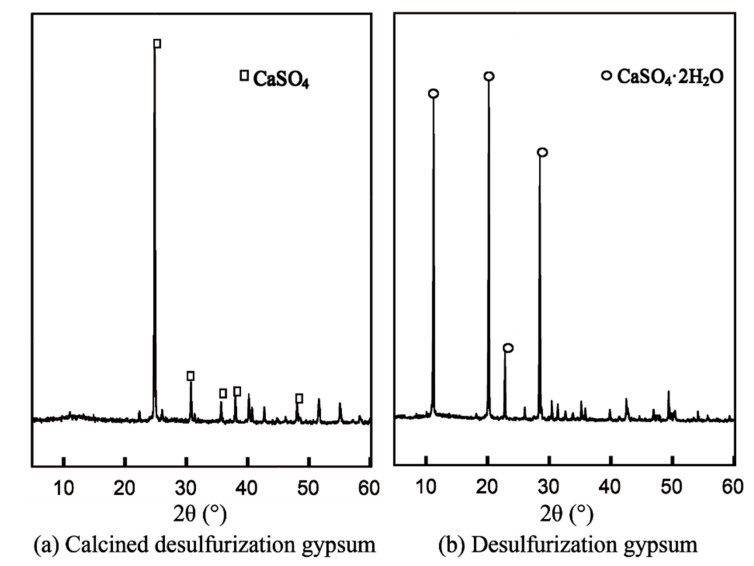
XRD of FGD gypsum.

The main mineralogical composition of FGD gypsum is CaSO_4_·2H_2_O, which can be clearly seen in Figure 2a. The decomposition of chemical-bound water of gypsum can occur when the temperature is higher than 120 °C and the two molecular H_2_O can be released totally within 400 °C. Therefore, the minerals observed in the calcined FGD gypsum consist of anhydrite as shown in Figure 2a. After thermal treatment, the composition was anhydrous calcium sulfate as can be seen in Figure 2b. The anhydrite can be totally decomposed into CaO and SO_3_ when the temperature is higher than 1300 °C in the absence of carbon [14]. However, even partial release of SO_3_ would leave a cavity in the particles with structural relaxation, which is able to improve the reactivity of CaSO_4_. At the same time, CaO had a certain catalytic effect on the hydration of anhydrite during the mixing with water. The more porous the particles’ surface, the more ions can penetrate into the particles’ interior, which can be utilized to favor the reactivity.

**Figure 3:**
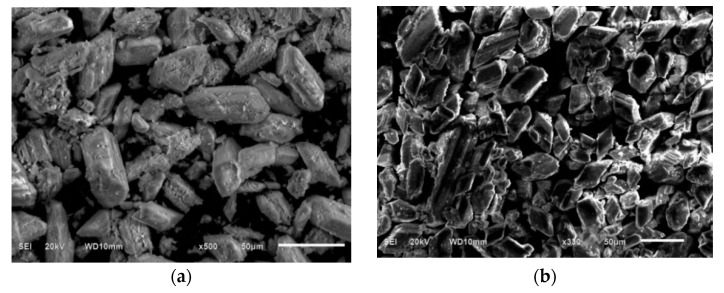
SEM of FGD gypsum. (**a**) Calcined FGD gypsum at 800 °C. (**b**) Raw FGD gypsum.

The microstructure of the raw and calcined FGD gypsum is shown in Figure 3. It can be seen that the calcination did not change the morphology but resulted in a difference in the crystal size. The calcination tends to increase the crystal size. Both of the images show a short column calcium sulfate crystal.

After reaching the designed testing age, the samples were firstly immersed in a high-purity ethanol solution to terminate hydration and then ground into powder. The XRD test was used to determine the hydration products.

### 2.2. Mixtures

Cement clinker minerals, mineral powder, and active calcium aluminate were dissolved in water to produce Al(OH)^4−^ and OH^−^ after water was added to the Portland cement-fly ash-calcined FGD gypsum-calcium aluminate composite quaternary cementitious system. The desulfurized gypsum also dissolved SO_4_^2−^ and Ca^2+^ rapidly. When these ions accumulate through diffusion, they react with each other to form ettringite. The reaction steps are as follows:

Al(OH)_4_^−^ + 2OH^−^ − [Al(OH)_6_]^3−^ (1)

[Al(OH)_6_]^3−^ + 3Ca^2+^ + 12H_2_O − {Ca_3_Al(OH)_6_·12H_2_O}^3+^ (2)

2{Ca_3_Al(OH)_6_·12H_2_O}^3+^ + 3 SO_4_^2–^ + 2H_2_O − {Ca_3_Al(OH)_6_·12H_2_O}_2_·(SO_4_)_3_·2H_2_O (3)

The theoretical ratio of sulfur to aluminum required for the reaction is 1.5:1 [15,16]. The mixtures of prepared mortar are given in Table 2. The calcined FGD gypsum and active calcium aluminate were used to substitute the fly ash, and the sulfur to aluminum ratio was set as 1.5:1 and 1:1 for F1 and F2, respectively. The sulfur content referring to the sulfur anhydride content in FGD gypsum and the alumina content were calculated from both fly ash and active calcium aluminate. The water binder (w/b) ratio was 0.5.

The corresponding neat pastes were also prepared using the same binders in Table 2. Hydrations of the pastes were terminated by alcohol, and ground to a fine powder, which can pass through a sieve of 80 um for microstructure determination.

### 2.3. Test Methods

#### 2.3.1. Setting Time

The setting time of samples was tested by using a Vicat apparatus, and the specific test procedure was based on BS EN 196-3-2016 [17]. Starting from the contact of the binder with the water, the initial setting time was recorded as the time it took for the test needle to be 4 mm from the bottom plate. The final setting time was recorded as the time until the test needle did not leave a circular impression on the surface of the pastes.

#### 2.3.2. Fluidity

The fluidity of samples was determined by conducting the flow table test following the ASTM C230 [18]. After filling the mold with mortar, the flow mold was lifted away, and the flow table was immediately dropped 25 times in 25 ± 1 s at a constant frequency. The average of two perpendicular spread diameters was measured.

#### 2.3.3. Compressive Strength

The mortar cubic (40 mm × 40 mm × 160 mm) was cast and cured in the molds for 1 day at 20 ± 2 °C, and then demolded and placed in a curing room (20 ± 2 °C, RH > 90%). The compressive strength was measured at ages of 1, 7, 28, 90, 180, and 360 days. Three replicates were tested for each mixture. 

#### 2.3.4. Volume Stability

The specimen dimensions were 40 mm × 40 mm × 160 mm for the free expansion ratio, with two test heads in the center of the two ends. The dimensions of the specimens for the limited expansion ratio were 40 mm × 40 mm × 160 mm, with one restraining cage in one specimen. After 1 day, the samples were demolded, and a length comparator was used to determine the dimension of the samples along the longitudinal axis. Then, the samples were transferred into a room with a temperature of 20 ± 2 °C and RH of 50 ± 5%. The samples were completely open to the environmental conditions. The free expansion ratio and limited expansion ratio of mortars were measured at the ages of 1, 3, 7, 14, 21, 28, 40, 60, 90, and 130 days. Three replicates were tested.

The 40 mm × 40 mm × 160 mm mortar samples were immersed in the water at the laboratory, where the temperature was controlled at 20 ± 2 °C and the relative humidity was over 95%. The appearance was checked every week to observe whether the deterioration of cracking, spalling, and warping occurred.

#### 2.3.5. Hydration Products

The hydration products of the hardened binder were determined by XRD analysis (Bruker AXS, Karlsruhe, Germany), whose X-ray diffractometer had monochromatic yielding Cu Ka radiation (voltage was 40 kV and current was 20 mA). The scanning scope was 5–80°.

#### 2.3.6. AFt Quantity Determination

The ethylene glycol-methanol solvent can extract AFt in cement hydration products with a high selectivity of 98%. Therefore, it is able to infer the AFt content in the composite by determining the content of Al_2_O_3_ in the extract [19,20,21,22]. The detailed process can be summarized as:

First, 1 g of hydration powder was added to an Erlenmeyer flask containing 20 mL of ethylene glycol-methanol extractant, and stirred for 2 to 3 h, and then filtered under vacuum. Then, 20% hydrochloric acid was used to dissolve the filtrate and dilute it to 50 mL with deionized water. Then, 5 mL of the filtrate were added to the acetic acid-ammonium acetate buffer solution to have a pH value of 5–6.

In total, 5 mL of EDTA (0.015 mol/L) standard solution were added to the above solution, and the solution was diluted to 20 mL, heated, and boiled for 3 min.

Five to six drops of xylenol orange solution were added to the above solution. When solution turned from colorless to yellow, and the color of the yellow solution did not change with the temperature, then the zinc sulfate standard solution (0.015 mol/L) was used to titrate. At the time indicator changed from yellow to red, the titration was stopped.

In total, 10 mL of EDTA standard solution (0.015 mol/L) were added to a 200 mL erlenmeyer flask, and then the acetic acid-ammonium acetate buffer solution was added to adjust the pH value to 5–6. Five to six drops of xylenol orange solution were added to the former solution and drop it back with zinc sulfate standard solution (0.015 mol/L). When the indicator changed from yellow to bright yellow to red, the titration was completed. The coefficient *K* of EDTA to ZnSO_4_ is shown in Equation (4):

(4) $$K=\frac{V\left({\mathrm{ZnSO}}_{4}\right)}{V\left(\mathrm{EDTA}\right)}$$

The AFt quantity (%) in the hardened paste:

(5) $$\mathrm{AFt}=\frac{C\left(\mathrm{EDTA}\right)\cdot \left[V\left(\mathrm{EDTA}\right)-K\cdot V\left({\mathrm{ZnSO}}_{4}\right)\right]\cdot M\left(\mathrm{AFt}\right)}{200\cdot M}$$

where *V*(EDTA) is the amount of EDTA solution added in the reaction (5 mL); *V*(ZnSO_4_) is the volume of ZnSO_4_ solution consumed in the titration process, mL; *M*(AFt) is the molecular mass of ettringite; *C*(EDTA) is the concentration of the EDTA solution (0.015 mol/L); *K* is the titration coefficient of EDTA solution to ZnSO_4_ solution; and *M* is the mass of the hydration product, g.

## 3. Results and Discussion

### 3.1. Workability and Compressive Strength 

The setting times of the paste and fluidity mortar are shown in Table 3 and Table 4. Compared with the fly ash prepared sample, the setting time was shortened with the addition of sulfate into the system since more ettringite was formed in the early period. Moreover, the setting time was slightly varied with increasing the sulfate content in the system. 

With the addition of calcined FGD, the fluidity of F1 and F2 was reduced compared with the neat fly ash prepared sample. This could be due to the relatively high water requirement of calcined FGD gypsum and the addition of calcium aluminate.

**Figure 4:**
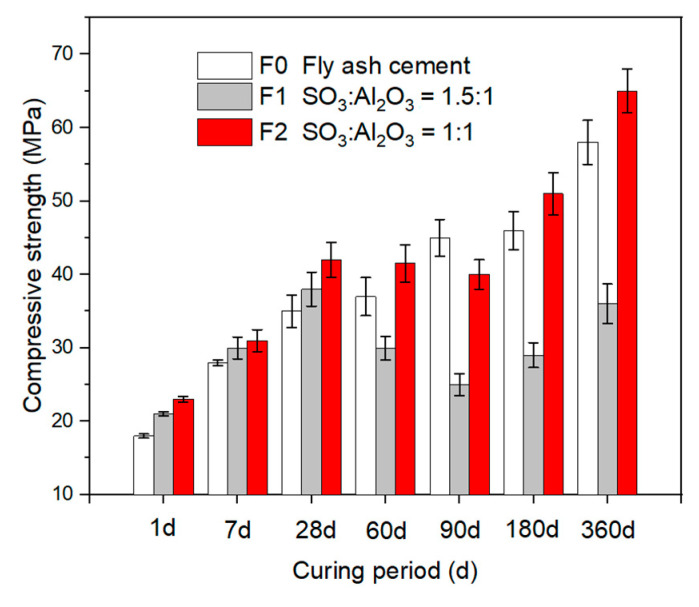
Compressive strength of specimens in steam curing.

After steam and standard curing, the development of compressive strength in relation to the age and sulfate content is shown in Figure 4. The addition of the sulfate and alumina phase, i.e., the FGD gypsum and calcium aluminate, improved the compressive strength at an early age. The compressive strength of F2 showed the highest compressive strength among all the samples. The compressive strength of F2 at 1, 28, and 360 days was 35%, 21%, and 11% higher than that of the control specimen. Further increasing the sulfate to alumina ratio was only beneficial for the compressive strength within the first month but led to a reduction at later ages. This indicates that the addition of an appropriate amount of sulfate and alumina could improve the strength, which is more remarkable at an early age.

### 3.2. Volume Stability

#### 3.2.1. Free and Limited Expansion

**Figure 5:**
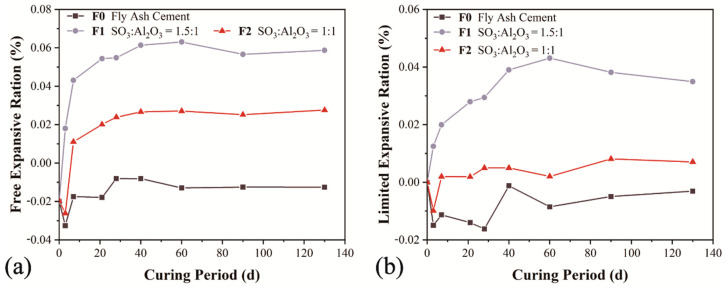
Free expansion (**a**) and limited expansion (**b**) ratio of specimens.

The effect of DFG gypsum and calcium aluminate on the free and limited expansion are shown in Figure 5a,b. It can be seen from Figure 5a that F1 and F2 expanded obviously within 40 days, especially in the first 14 days. Using a high sulfate to alumina ratio tends to promote expansion. The higher the SO_3_ content, the more ettringite could be formed and a larger expansion can be expected. Regarding the limited expansion in Figure 5b, only a remarkable expansion could be observed when the sulfate to aluminate ratio is 1.5, and no significant expansion was observed in F2.

#### 3.2.2. Long-Term Immersion Expansion

**Figure 6:**
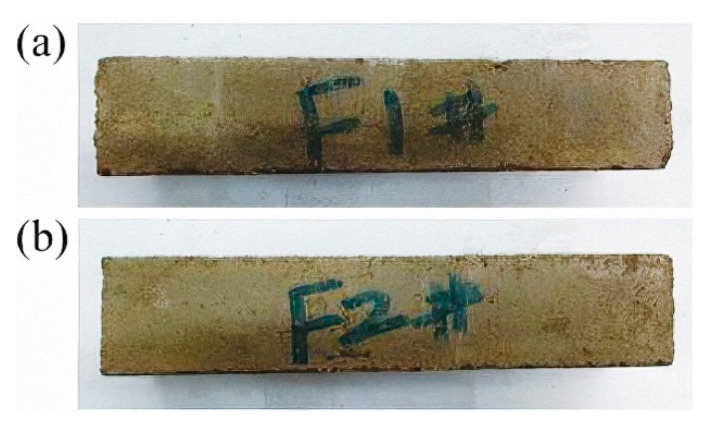
Images of F1 (**a**) and F2 (**b**) specimens after 360 days of hydration.

In order to further verify whether the ettringite formed from the reaction between calcined FGD gypsum and calcium aluminate could cause expansion, mortar specimens were immersed in water for one year. Figure 6a,b shows the image of F1 and F2 cured in water until 360 days. No signs of cracking, spalling, and warping appeared on the surface.

The addition of calcined FGD gypsum and calcium aluminate did not exert adverse effects on the stability of the cementitious system. The above phenomenon can be favored by the expansion stress calculation:

σ = μ∗Ε∗ε(6)

where σ is the expansion stress; μ is the reinforcement ratio, and the value of 0.78% was applied in this study; Ε is the elastic modulus of limit reinforcement; and ε is the limit expansion ratio at the determined age.

According to Equation (6), specimen expansion stress can be calculated by the measured limited expansion ratio. The tensile strength was 7~9% of the compressive strength commonly (and the intermediate value of 8% was adopted in this work). The final results are given in Table 4. It can be seen that the expansion stress of F1 and F2 was lower than the tensile strength of cementitious mortar, so there is no risk of expansion for the designed two systems.

### 3.3. Kinetics of Ettringite Formation

**Figure 7:**
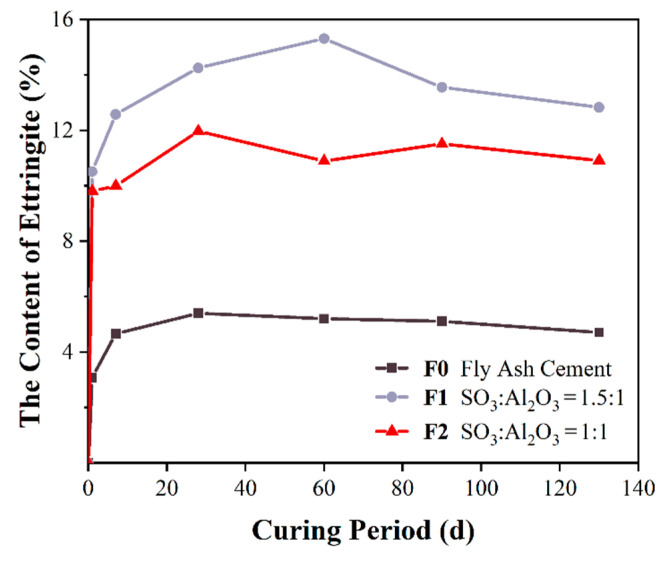
Content of ettringite of samples.

Figure 7 shows the ettringite content of the corresponding pastes of F1 and F2 at each testing age. There was a rapid growth of ettringite in the first 14 days, and the increase was gentler from 14 to 28 days, and reached a stable value or even dropped slightly subsequently. It is clearly seen that the S1 showed the highest ettringite content for all determined ages and the control sample showed the least value, which means that a small amount of aluminate cement can provide aluminate ions to promote the formation of early ettringite, and improve the early strength. The content of ettringite reached its peak at 60 and 28 days for F1 and F2. Due to the consumption of aluminate ions, the generation rate of ettringite decreased and the content of ettringite in the system tended to be stable in the later stage. Therefore, there is no risk of DEF occurring, and the expansion stress is less than the tensile strength (Table 5), and the specimens have no risk of cracking. This finding is consistent with the free expansion and limited expansion results as shown in Figure 5. The relatively slow dissolution of FGD gypsum affects the concentration of sulfate ions in the pore solution and thus limits the formation rate of AFt, which is directly related to the expansion and mechanical properties [23,24,25].

### 3.4. Hydration Products

**Figure 8:**
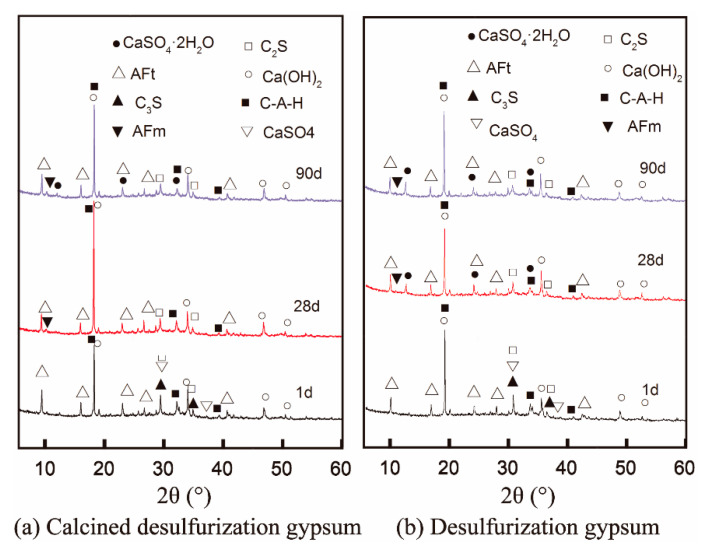
XRD pattern of sample F1 and F2 in standard curing for 1, 28, and 90 days.

Figure 8a,b show the XRD patterns of samples at 1, 28, and 90 days. The results show that the hydration products are mainly composed of AFt, C-A-H, and Ca (OH)_2_. Additionally, a small amount of AFm appeared at 28 days, suggesting that part of the ettringite was transformed into AFm in the later age. Moreover, the characteristic peak of anhydrite disappeared at 28 days The characteristic peak of gypsum is presented since the crystal size of gypsum is larger than that of anhydrite and the transformation could lead to an expansion in the volume [8,26,27].

Combined with the evolution of ettringite and hydration products, the drop of mortar strength for F1 from 28~90 days can be contributed by the following two reasons: on the one hand, the ettringite content increases continuously after 28 days. When the hardened paste has limited space for the precipitation of AFt, the excessive expansion stress could lead to cracking of the hardened pastes [28,29]. On the other hand, the transformation of fine anhydrite to large gypsum crystals would also risk the integrity of the pastes. Both of these reasons could cause some damage to the hardened system. Considering the composition of F1, the excessive sulfate to alumina ratio could favor the stability of ettringite and promote the deposition of gypsum at a later age. This is the main reason for the compressive strength reduction observed from 28 to 90 days.

## 4. Conclusions

Owing to the promotion of the early strength development of fly ash blended cement for precast concrete manufacturing, fly ash was partly substituted by calcined FGD gypsum and calcium aluminate was introduced. The effect of the sulfur to aluminum ratio on the compressive strength, volume stability, and evolution of hydration products was determined systematically.

In the Portland cement-fly ash-calcined FGD gypsum-calcium aluminate composite quaternary cementitious system, an appropriate sulfate to aluminate ratio is beneficial for the development of compressive strength and volume stability. Compared with the fly ash blended control sample, the setting time was shortened and the fluidity was reduced slightly. Both the free expansion and limited expansion of calcined FGD gypsum and calcium aluminate blended specimens showed growth in the linear dimension, and most of the expansion occurred in the first 14 days.

Due to the excessive sulfate to aluminate ratio, the continuous formation of ettringite and the transformation of anhydrite into gypsum is the main reason for the remarkable expansion and compressive strength reduction at a later age for F1. Regardless of the sulfate to aluminate ratio, no cracks were observed in all specimens even after being immersed in water for one year.

A general expansion stress calculation method was proposed. In the composite cementitious system, there was no risk of cracking since the calculated expansion stress was lower than the tensile strength. 

## Figures and Tables

**Table 1:** The chemical composition of P.II.52.5 Portland cement, FGD gypsum, and fly ash (wt/%).

Item	CaO	SiO_2_	Al_2_O_3_	Fe_2_O_3_	MgO	SO_3_	TiO_2_	K_2_O
Portland cement	61.3	20.8	6.3	3.1	1.0	2.3	0.3	0.9
FGD gypsum	39.5	3.3	0.9	0.6	1.2	53.0	\	\
Fly ash	4.8	51.6	22.6	14.9	1.7	1.0	\	\

**Table 2:** Mix proportion of cement-fly ash-FGD-active-calcium aluminate mortar (wt/%).

No.	Cement	Sand	FGD	Fly Ash	Active-Calcium	SO_3_	SO_3_:Al_2_O_3_	w/b
F0	17.5	75.0	0	7.5	0	0	/	0.5
F1	17.5	75.0	1.9	5.0	0.6	4.4	1.5:1	0.5
F2	17.5	75.0	1.6	5.0	0.9	3.7	1:1	0.5

**Table 3:** Setting time of specimens.

Setting Time	Initial Setting Time (min)	Final Setting Time (min)
F1(SO_3_:Al_2_O_3_ = 1.5:1)	138	218
F2(SO_3:_Al_2_O_3_ = 1:1)	153	225
F0	204	235

**Table 4:** Mortar fluidity of specimens.

	F1(SO_3_:Al_2_O_3_ = 1.5:1)	F2(SO_3:_Al_2_O_3_ = 1:1)	F0
Mortar fluidity (mm)	135	150	160

**Table 5:** Expansion stress and tensile strength of specimens.

		3d	7d	28d	40d	60d	90d	180d
F1(SO_3_:Al_2_O_3_ = 1.5:1)	Expansion stress	0.23	0.29	0.59	0.53	0.60	0.61	0.62
	Tensile strength	1.64	2.40	3.00	2.80	2.40	2.00	2.45
F2(SO_3_:Al_2_O_3_ = 1:1)	Expansion stress	−0.24	−0.16	0.23	0.31	0.29	0.46	0.36
	Tensile strength	1.79	2.42	3.38	3.34	3.34	3.31	4.03
F0	Expansion stress	−0.59	−0.47	v0.28	0.09	0.03	0.14	0.11
	Tensile strength	1.33	2.24	2.50	2.71	3.10	3.51	3.64

## Data Availability

Not applicable.
